# Editorial: Animal reproduction under extreme environments

**DOI:** 10.3389/fvets.2024.1355376

**Published:** 2024-03-01

**Authors:** Víctor H. Parraguez, Antonio González-Bulnes

**Affiliations:** ^1^Facultad de Ciencias Veterinarias y Pecuarias, Universidad de Chile, Santiago, Chile; ^2^Facultad de Ciencias Agronómicas, Universidad de Chile, Santiago, Chile; ^3^Facultad de Veterinaria, Universidad Cardenal Herrera-CEU, CEU Universities, Valencia, Spain

**Keywords:** reproduction, harsh environment, melatonin, GnRH, colostrum in underfed pregnancies

Population growth is exerting a significant influence on both the expansion of human settlements and the need to produce more food and energy. This has led to an increased demand for cultivable lands, either for production of vegetal foods or biomass for energy production. Consequently, there is a heightened pressure on animals, especially livestock, to be relocated to territories with harsh environmental conditions, where agriculture may be impractical and sustaining human and animal life becomes more challenging. On the other hand, poor rural populations, who are important owners of farm animals, usually live in marginal territories and, in general, depend on their animals as the main source of food and income. Therefore, under this scenario, a better understanding of how the exposure of animals to different extreme environmental conditions (cold, heat, wind, hypoxia, poor nutritional resources, among others) affects their reproductive process becomes crucial, as reproduction success is one of the bases of animal production and settlement.

In line with these challenges, our Research Topic contemplates a topic of vital importance for professionals advising farm animal owners or livestock policymakers. Notably, an interesting contribution to our Research Topic was a work done on swamp water buffaloes, a species playing a pivotal role in the agricultural economies of subtropical countries in Southeast Asia, providing meat, milk, leather, and draft power. In these animals, reproductive activity decreases during the months of longer photoperiod, associated with heat stress. On the contrary, during the months of short photoperiod, where environmental temperature decreases and melatonin concentrations are higher, various reproductive parameters improve. The authors tested the effects of the administration of melatonin during heat stress season, administered by i.m. injection, 1 day before or on days−3,−2 and−1 of the start of an Ovsynch protocol to synchronize the estrus of multiparous buffaloes, on variables associated with ovarian activity and fertility after artificial insemination, as well as on traits of the milk quality and the immunological status. Interestingly, the results showed that the administration of a dose of melatonin improved follicular growth, ovulatory rate and estrous response. Additionally, melatonin, in both forms of administration, increased pregnancy rate and plasma concentration of IgM, and decreased the count of somatic cells in milk. The authors concluded that the administration of melatonin at the beginning of synchronization protocols is a good tool to improve reproduction and health aspects of milk in swamp water buffaloes during the low breeding season (details in Abulaiti, Nawaz et al.).

Another interesting contribution to our Research Topic discusses the importance of embryonic losses after artificial insemination with various heat synchronization protocols, as a main cause of reduced fertility during the low reproductive seasons in crossbreed buffaloes. These losses seem to be associated with a deficiency in P_4_ concentrations, so in this article it was proposed to improve progestational competence through the strategic application of different doses of GnRH on day 20 after AI. The results showed that the application of 200 μg of GnRH favored the development of the corpus luteum and, as a consequence, the plasma concentrations of P_4_. Although no significant differences were detected between animals without GnRH or with lower doses of the hormone, buffaloes that received 200 μg of GnRH presented higher absolute value of pregnancy rate and lower embryo-fetal losses until day 90 after AI. The authors suggested that this hormonal therapy can be a good tool to improve the fertility of buffaloes during the season of low reproductive efficiency. However, further studies are needed to confirm these preliminary results (details in Abulaiti, Naseer et al.).

Our Research Topic also addressed a topic relevant to sheep, acknowledging their widespread distribution in various territories under extreme conditions, where food scarcity is a common challenge. However, even in environments with nutritional restriction, twin pregnancies are frequent in sheep. Compared to singletons, twin lambs are born not only with lower weight, increasing their probability of getting sick or dying in the early postpartum, but also, multiple gestation occurring in as nutrient restricted environment, may affect other maternal variables which influences animal survival and post-natal performance. The quality and quantity of maternal colostrum and its consumption by lambs is essential for thermoregulation and to achieve an adequate immune status. In this study, the yield and nutritional quality of colostrum, as well as its IgG content were compared between sheep that gave birth single and twin lambs, whose pregnancies took place in environments of nutritional restriction. Newborn blood IgG and glucose was also assessed. The main results showed that, under these conditions, there were no differences in the amount of colostrum delivered by the mothers, which puts twin lambs at a disadvantage, as they may have access only to a half of the colostrum amount a single-born lamb may consume. The colostrum of ewes that gave birth to twins had lower lactose, protein and IgG concentrations compared to those that gave birth to singletons. Both the composition and the amount of colostrum consumed by the twins negatively impacted blood glucose and IgG concentrations during the first hours after birth, which meant a high mortality of twins in the first month of life (details in Turin et al.).

In conclusion, we are confident that this Research Topic will contribute significantly to enhance the efficiency of reproductive and productive processes, as well as the reproductive health of animal species, crucial to human populations inhabiting territories with extreme environments.

## Author contributions

VP: Writing – original draft. AG-B: Writing – review & editing.

